# Die Berücksichtigung von Gesundheit in deutschen (Sport‑)Curricula

**DOI:** 10.1007/s11553-023-01028-5

**Published:** 2023-04-24

**Authors:** Katharina Pöppel, Greta Webner

**Affiliations:** Institut für Sportwissenschaft, Sport und Erziehung, Carl von Ossietzky Universität Oldenburg, Ammerländer Heerstraße 114–118, 26129 Oldenburg, Deutschland

**Keywords:** Curricula, Sportunterricht, Health Literacy, Gesundheitsförderung, Bildung, Curricula, Physical education, Health literacy, Health promotion, Learning

## Abstract

**Hintergrund:**

Gesundheit stellt in Deutschland kein Schulfach an allgemeinbildenden Schulen dar und dennoch soll schulische Bildung einen Beitrag zur Gesundheitsförderung leisten. Die Auslegung dieser Zielsetzung reicht von einer gesundheitsförderlichen Wirkung durch Bewegung per se bis zum Anspruch eines reflexiven Gesundheitsverständnisses, das sich durch das Konstrukt Health Literacy (HL) beschreiben lässt.

**Ziel der Arbeit und Methode:**

Zur Prüfung, inwieweit ein reflexives Gesundheitsverständnis in der schulischen Bildung Deutschlands gemäß HL-Ansatz angelegt ist, werden 33 gegenwärtig gültige Kerncurricula allgemeinbildender Schulen in Deutschland inhaltsanalytisch ausgewertet.

**Ergebnisse:**

Vor allem der Biologie- und der Sportunterricht ab dem Primarbereich zielen auf eine Förderung gesundheitsbezogener Kompetenzen ab. Die Analyse ergibt, dass eine sportmotorisch ausgerichtete und somit handlungsorientierte Förderung von HL im Fach Sport dominiert. Darüber hinaus soll der Sportunterricht nur in wenigen Bundesländern gleichermaßen zum Verstehen, Auswerten, Kommunizieren und Entscheiden hinsichtlich dieser Inhalte anregen.

**Schlussfolgerung:**

Es bleibt unklar, ob gesundheitsbezogene Inhalte im Zentrum des Unterrichts stehen sollen oder der Gesundheitsbezug lediglich als Etikett dient. Dennoch birgt v. a. der Sportunterricht das Potenzial, Heranwachsende frühzeitig und umfassend für gesundheitsbezogene Themen zu sensibilisieren.

## Abstract (en)

**Background:**

In Germany, health is not a subject in general schooling, yet school education is supposed to contribute to health promotion. The interpretation of this objective ranges from a health-promoting effect through exercise to the claim of a reflexive understanding of health that can be described by the construct of health literacy (HL).

**Objective and methods:**

In order to examine the extent to which a reflexive understanding of health is applied in school education in Germany according to the HL approach, 33 currently valid curricula of general education schools in Germany are evaluated by content analysis.

**Results:**

In particular, biology and physical education classes from the primary level onward aim to promote health-related competencies. The analysis shows that a sport motoric and, thus, exercise-oriented promotion of HL dominates in physical education. Furthermore, only in a few federal states is physical education intended to stimulate the understanding, evaluation, communication, and decision-making concerning these contents.

**Conclusion:**

It remains unclear whether health-related content should be the focus of instruction or whether the health reference merely serves as a label. Nevertheless, physical education especially has the potential to sensitize adolescents early and comprehensively to health-related topics.

## Hintergrund

Die Coronaviruspandemie (COVID-19-Pandemie) führte u. a. dazu, dass eine verstärkte Betrachtung von Gesundheit und Schule und somit auch schulischer Bildung erfolgte [11]. Ein besseres Gesundheitsverständnis ist altersunabhängig nötig, um beispielsweise der Verbreitung von Erkrankungen entgegenzuwirken oder um ein gesundes Leben zu führen. Somit ist es bereits für junge Menschen zentral, die Qualität gesundheitsbezogener Informationen einschätzen und nutzen zu können.

Gesundheit stellt kein eigenständiges Schulfach an allgemeinbildenden Schulen dar [21]. Dennoch finden sich gesundheitsbezogene Anknüpfungen beispielsweise im Sportunterricht, dessen Bedeutsamkeit über reines Sporttreiben hinausgeht [18]. Der vorliegende Artikel verfolgt daher das Ziel zu analysieren, welchen Beitrag schulische Bildung und hierbei insbesondere der Sportunterricht zur Förderung eines kompetenten, gesundheitsförderlichen Verhaltens von Kindern und Jugendlichen leisten kann.

## Health Literacy bei Kindern und Jugendlichen

Gesundheit ist ein komplexes Konstrukt, das weit über eine Auseinandersetzung mit dem Krankheitsbegriff hinausreicht [8]. Eine verbreitete ganzheitliche Modellvorstellung legt ein Zusammenspiel von somatischen, psychischen und sozialen Komponenten zugrunde [7]. Betrachtet man dieses biopsychosoziale Gesundheitsverständnis, kann man schlussfolgern, dass sich der psychologische Anteil im Sinne von Kognitionen und Verhalten leichter beeinflussen lassen sollte als der somatische Anteil im Sinne einer Disposition oder der soziale Anteil, der sich beispielsweise auf den sozioökonomischen Status einer Person beziehen lässt. Weiterhin unterscheidet man ein salutogenetisches Gesundheitsverständnis, welches primär auf eine Stärkung von Ressourcen und Schutzfaktoren abzielt [1].

Ein Konstrukt, das sich auf die Fähigkeit einer Person, Gesundheitsinformationen zu verstehen, einzuschätzen, anzuwenden und reflektierte Urteile hinsichtlich der Gesundheitsversorgung, Krankheitsprävention oder Gesundheitsförderung zu treffen bezieht, ist Health Literacy (HL; [22]). Das dargelegte Begriffsverständnis zielt auf Erwachsene ab und erscheint nur in Ansätzen kompatibel mit der Lebenswelt Heranwachsender [4]. Bröder et al. ([5, S. 11] übersetzt aus dem Englischen) entwickelten eine Arbeitsdefinition von HL für Jugendliche, die „umfasst, wie gesundheitsbezogene, multimodale Informationen aus verschiedenen Quellen abgerufen, verstanden, bewertet und kommuniziert werden und zur Entscheidungsfindung in verschiedenen Situationen in Gesundheits-(Versorgungs‑)Settings und Kontexten des täglichen Lebens genutzt werden, wobei soziale, kognitive und rechtliche Abhängigkeiten berücksichtigt werden.“ HL wird als Ergebnis einer gezielten Gesundheitserziehung gesehen und stellt eine wichtige soziale Ressource dar, die auch Prävention und körperliche Aktivität umfasst [27]. Schulischer Bildung kommt somit eine besondere Bedeutung zu.

International wird beobachtet, dass ein niedriger Bildungsstand der Eltern mit einer niedrigen HL ihrer Kinder einhergeht [10]. Gerade im Jugendalter treffen zwei Komponenten aufeinander: eine gesteigerte Tendenz zu Risikoverhalten [2] und die eigene Ausprägung an HL, die das gesundheitliche Risikoverhalten prägt [20]. Hinzu kommt, dass gesundheitlich riskanten Verhaltensweisen (z. B. Rauchen oder übermäßiger Alkoholkonsum) aus Perspektive eines jungen, gesunden Körpers eine geringere langfristige Bedeutsamkeit beigemessen wird [2]. Demnach ist eine frühe professionelle Initiierung von Lehr-Lern-Prozessen zur Steigerung von HL umso wichtiger, insbesondere im Bereich der Sekundarstufe (Sek.) I und somit in den Altersgruppen von 10-16 Jahren.

## Schulische Bildung und Gesundheitsförderung

Schulische Bildung wird auf Bundeslandebene über die Curricula der einzelnen Schulfächer festgelegt, die Lerninhalte und avisierte Kompetenzzuwächse spezifizieren. Curricula können somit als Filter verstanden werden, was gegenwärtig für Kinder und Jugendliche als bildungswert erachtet wird [24]. In Deutschland existiert kein spezifisch auf Gesundheit ausgerichtetes Schulfach an allgemeinbildenden Schulen. Dennoch wird beispielsweise in den Ausführungen zum Auftrag des Schulsports explizit auf Gesundheitsförderung als Zielsetzung verwiesen [6]. Demnach scheint gerade der Sportunterricht ein Bildungspotential zur Steigerung von HL zu bieten. Für Sportlehrkräfte ist es herausfordernd, den Bereich Gesundheit didaktisch und methodisch so umzusetzen, sodass er für Schüler*innen relevant wird [18].

Im Zuge der Kompetenzorientierung bilden v. a. das fachdidaktische Prinzip der Mehrperspektivität in Kombination mit sechs pädagogischen Perspektiven die Grundlage zahlreicher aktuell gültiger Kerncurricula des Fachs Sport in Deutschland [13, 25]. Hierbei wird explizit auf die Bereiche Gesundheit, Verbesserung körperlicher und geistiger Fitness sowie Entwicklung von Gesundheitsbewusstsein verwiesen. Vor allem die Entwicklung einer gesundheitsbezogenen Handlungsfähigkeit steht im Vordergrund, die aus der Reflexion eigener Erfahrungen resultieren soll.

## Zielsetzung der Untersuchung

Da schulische Bildung theoretisch über ein großes Potenzial zur Förderung von HL bei Kindern und Jugendlichen verfügt, zielt die Studie auf eine curriculare Analyse des Ausmaßes gesundheitsförderlicher Themenbereiche gemäß HL-Verständnis in den Lehrplänen allgemeinbildender Schulen ab. Hierbei wird v. a. ein Fokus auf den Bildungsbeitrag des Sportunterrichts gelegt. Die Fragestellung greift drei Bereiche auf: 1) Wie konstituiert sich der Beitrag schulischer Curricula zur Förderung von HL unter besonderer Berücksichtigung des Sportunterrichts? 2) Welche Altersgruppen werden mit HL-bezogenen Themen adressiert? 3) Welches Gesundheitsverständnis und welche Dimensionen von Gesundheit werden angesprochen?

## Methodik

Im Vorfeld der Analyse wurde eine Präregistrierung beim Center for Open Science vorgenommen [19]. Mittels qualitativer Inhaltsanalyse [14] erfolgte eine Untersuchung schulischer Curricula auf HL-Lehrinhalte, die auf eine Systematisierung der Inhalte und eine Reduktion von Komplexität abzielte. In die Analyse gehen 33 Curricula ein: Die Curricula für Sportunterricht im Bereich Sek. I aller 16 deutschen Bundesländer, die gegenwärtig gültigen Curricula aller Unterrichtsfächer der Sek. I des Bundeslands Nordrhein-Westfalen als bevölkerungsreichstes deutsches Bundesland, sowie ergänzend die nordrhein-westfälischen Sportunterrichtscurricula für den Bereich der Primarstufe und der Sek. II. Falls für den Sek.-I-Bereich mehrere, nach Schulform differenzierte Curricula vorlagen, wurde hierbei das gymnasiale Curriculum gewählt.

In einem deduktiven Ansatz wurden aus der HL-Definition von Bröder et al. [5] Kategorien und Unterkategorien abgeleitet und im Sinne einer formativen Reliabilitätsprüfung ergänzt durch Perspektiven des Australian Curriculum for Health and Physical Education [3], da die Förderung von HL eine der Schlüsselkomponenten des Curriculums darstellt (Tab. 1). Kodierregeln und Ankerbeispiele wurden hierbei als operationalisierte Definition der Kategorie zusammengefasst. Die Durchführung der Inhaltsanalyse erfolgte mithilfe der Software MAXQDA 2020 [28] zur Strukturierung der Kategorien.

**Tab. 1:** Kategoriensystem der inhaltsanalytischen Untersuchung deutscher Schulcurricula auf HL-Inhalte (Health Literacy) orientiert an Bröder et al. [9], ergänzt durch Ausführungen des australischen Curriculums für Sport- und Gesundheitserziehung [20]

Initiales Kategoriensystem	Operationalisierte Definition der Kategorie
Umgang mit gesundheitsbezogenen Informationen unter Berücksichtigung sozialer, kognitiver und rechtlicher Abhängigkeiten:	
Zugang zu Informationen (Fertigkeit)	Selbstständiges Recherchieren bzw. Abrufen von gesundheitsbezogenen Informationen. Dies impliziert Wissen, wo man Informationen sucht bzw. findet, ohne die Qualität der Informationen zu bewerten (z. B. selbstständig entnehmen etc.)
Verstehen von Informationen (Wissen)	Korrektes Verständnis von gesundheitsbezogenen Informationen und demnach gesundheitsbezogener Wissensaufbau. Beinhaltet Wissenstransfer, im Sinne der Anwendung von (neuen) Informationen auf veränderte Umstände (z. B. unterscheiden, (er)kennen, begreifen, aneignen)
Auswertung von Informationen (Fertigkeit)	Analyse und (kritische) Bewertung von gesundheits-bezogenen Informationen aus verschiedenen Quellen(-arten) zur Erhaltung und Förderung der eigenen Gesundheit. Dies impliziert Transfer. Beinhaltet die Auswahl von zuverlässigen/glaubwürdigen und relevanten Informationen bzw. Quellen und ist inhaltsspezifisch (z. B. beurteilen, begründen, einordnen, deuten, reflektieren, einschätzen)
Kommunikation von Informationen (Fertigkeit)	Korrekte verbale Wiedergabe und Weitergabe von gesundheitsbezogenen Informationen (z. B. beschreiben, benennen, erläutern, verbalisieren, austauschen)
Informierte Entscheidungsfindung (Fertigkeit)	Intelligente Entscheidungen (individuell) in Bezug auf die Erhaltung oder Förderung der Gesundheit in einer Vielzahl von Umgebungen treffen, *einschließlich herausfordernder Szenarien*. [z. B. auswählen, für sich beurteilen]
Gesundheitsfördernde Maßnahmen (Handlung)	Durchführen von gesundheitsressourcenorientierten Handlungen – sowohl fremd- als auch selbstgesteuert. *Diese Handlungen können darauf abzielen, das Wohlbefinden zu steigern oder anderen zu helfen (z.* *B. Erste Hilfe leisten).* (z. B. aus/durchführen, sich belasten, anwenden, bewältigen, erfahren, verbessern, demonstrieren)
Differenzierte Betrachtung persönlicher Ausgangslagen	Berücksichtigung der persönlichen Ausgangslage (z. B. sozioökonomischer Status der Eltern, Milieuspezifika, soziales und kulturelles Kapital, Alters- und Entwicklungsspezifika) sowohl in der Betrachtung (Blick von außen = passiv) als auch in der Gestaltung der Lernumgebung (= aktiv)
Berücksichtigung vielschichtiger soziokultureller Lernprozesse	Adressierung des individuellen Erfahrungshintergrunds

Im Rahmen der Inhaltsanalyse wurden ausschließlich Curricula berücksichtigt, die gesundheitsbezogene Themenbereiche spezifizieren. Die selektive Reduktion manifester Materialinhalte erfolgte mittels Stichwortsuche und unter Berücksichtigung der nachfolgenden Begriffe bzw. Wortstämme in den jeweiligen Dokumenten: Gesund*, Leistung*, Wohl*, Salutogenese, Krank* oder Beschwerde*. Darauf aufbauend erfolgt eine Untersuchung der selektierten Kerncurricula durch eine qualitative Inhaltsanalyse [14], welche auch quantitative Elemente enthält. Das Vorgehen der Inhaltsanalyse orientiert sich an Krippendorffs [12] Empfehlungen zur Reduktion von Fehlern im Selektions- und Kodierprozess. Der unabhängige Kodierprozess erfolgte, indem ein Kodierer das gesamte Material entsprechend der zuvor definierten Kategorien bearbeitete (390 kodierte Textelemente), während ein zweiter unabhängiger Kodierer Ausschnitte des Materials analysierte (37,4 %). Hierbei handelte es sich um 129 Textelemente aus vier Curricula sowie 17 Elemente hinsichtlich derer der erste Kodierer eine Rückversicherung bezüglich der Einschätzung angezeigt hatte. Im Rahmen der Kodierung wurde jedes HL-bezogene Textelement einer Kategorie zugeordnet. Mehrfachkodierungen eines Elements waren nicht zulässig, allerdings konnte ein Satz aus mehreren Textelementen bestehen, die verschiedenen Kategorien zuordnet wurden. Das zuvor aus der Theorie abgeleitete Kategoriensystem hätte hierbei bei Bedarf um weitere HL-Inhaltskategorien ergänzt werden können, sofern eine Notwendigkeit seitens der Kodierer angezeigt wurde. Von dieser Möglichkeit wurde kein Gebrauch gemacht. Demnach konnte das initiale Kategoriensystem als vollständig angesehen werden. Die Interrater Reliabilität der vorliegenden Kodierung weist mit Krippendorff’s *α* = 0,881 (bootstrap10000 95 %-Konfidenzintervall [KI]: 0,817, 0,936) eine sehr gute Übereinstimmung der beiden Kodierer auf [12]. Vorliegende Unterschiede in der Kodierung wurden im Vorfeld der Inhaltsanalyse theoriegeleitet konsensvalidiert. Zehn Kodierungen wurden von den beiden Kodierern aufgrund einer zu großen inhaltlichen Entfernung zum Zielkonstrukt HL von der weiteren Analyse ausgeschlossen. Die finale Stichprobe beinhaltet 380 kodierte Inhaltselemente mit HL-Bezug.

## Ergebnisse

### Beitrag schulischer Curricula zur Förderung von HL

Die Inhaltsanalyse deutscher Curricula ergibt, dass v. a. der Sportunterricht einen Beitrag zur Förderung von HL bei Schüler*innen leisten kann (Tab. 2). In der Gesamtschau aller Bundesländer wird ein heterogenes Bild im Vergleich der HL-Anteile der Sportcurricula (Sek. I) von drei kodierten (Hamburg, [9]) bis 89 kodierten (Thüringen; [26]) Textelementen mit HL-Bezug deutlich. Weiterhin zeigt sich eine Dominanz konkreter anzustrebender Handlungen und somit des HL-Aspekts gesundheitsfördernde Maßnahmen (35,3 %, *n* = 134). Die kodierte HL-Komponente erscheint hierbei häufig als inhaltliche Rahmung zu den formulierten physischen Kompetenzen, z. B. „Die Schülerinnen und Schüler können ein gesund-funktionales Muskeltraining (z. B. als Zirkeltraining) unter Berücksichtigung der individuellen Belastungswahrnehmung sachgemäß durchführen“ (Nordrhein-Westfalen, [16]). Die zweithäufigste Kodierung entfällt auf die Kategorie Verstehen von Informationen (27,6 %, *n* = 105), die in vier Bundesländern dominiert (Saarland: 52,9 %, *n* = 9; Sachsen: 47,5 %, *n* = 19; Schleswig-Holstein: 45,0 %, *n* = 9; Sachsen-Anhalt: 44,4 %, *n* = 24). Zu dieser Kategorie zählen Kompetenzformulierungen, die auf eine Aneignung von gesundheitsbezogenem Wissen abzielen, z. B. „Grundlegende Wissensbestände: […] Zusammenhänge zwischen einer ausgewogenen Ernährung, körperlicher Fitness, gesunder Körperhaltung und geistigem Leistungsvermögen.“ (Sachsen-Anhalt, [15, S. 26]). Eine umfassendere Abdeckung des Konstrukts HL im Sinne eines Zusammenspiels von Handlungen, reflexiven und kommunikativen Anteilen wird nur in Thüringen und in Ansätzen in Nordrhein-Westfalen deutlich: „[…] die Bedeutung und Wirkung von Bewegung und Sport für die eigene Gesundheit erkennen, umsetzen und reflektieren“ (Thüringen, [26, S. 9]). Auffällig ist weiterhin, dass in den Formulierungen der Curricula weitgehend auf eine eigenständige gesundheitsbezogene Informationsbeschaffung verzichtet wird (Zugang zu Informationen). Eine differenzierte Betrachtung von persönlichen Ausgangslagen (z. B. sozioökonomischer oder kultureller Hintergrund) wird zwar in den allgemeinen Formulierungen der Curricula als globale Orientierung im Vorfeld der Kompetenzspezifikation benannt, allerdings im Bereich der konkreten Kompetenzformulierungen nicht weiter aufgegriffen. Gleiches gilt für eine nicht-vorhandene Berücksichtigung soziokultureller Lernprozesse.

**Tab. 2:** Anteil an HL-Textelementen (Health Literacy) in den deutschen Sportcurricula der Sek. I

	Zugang	Verstehen	Auswerten	Kommunikation	Entscheidung	Handlung	Ausgangslage	Soziokul.Lernen	Ges
BW (2016)	–	1	6	7	2	10	–	–	*26*
BY (2017)	–	7	3	6	3	20	–	–	*39*
BE/BB (2006)	–	1	–	1	1	1	–	–	*4*
HB (2006)	–	1	3	3	3	8	–	–	*18*
HH (2011)	–	1	–	–	–	2	–	–	*3*
HE (2011)	–	3	1	3	2	5	–	–	*13*
NI (2017)	–	3	–	1	–	4	–	–	*8*
NW (2019)	1	5	6	4	2	7	–	–	*25*
MV (2010)	–	7	2	–	–	9	–	–	*18*
RP (1998)	–	1	–	–	–	5	–	–	*6*
SL (2008)	–	9	2	–	1	5	–	–	*17*
SN (2009)	–	19	5	2	–	14	–	–	*40*
ST (2016)	–	24	9	4	3	14	–	–	*54*
SH (2015)	1	9	3	1	1	5	–	–	*20*
TH (2016)	–	14	21	12	15	26	1	–	*89*
Ges	*2* *(0,5* *%)*	*105* *(27,6* *%)*	*61* *(16,1* *%)*	*44* *(11,6* *%)*	*33* *(8,6* *%)*	*134* *(35,3* *%)*	*1* *(0,3* *%)*	*0*	*380*

*BW* Baden-Württemberg, *BY* Bayern, *BE/BB* Berlin und Brandenburg, *HB* Bremen, *HH* Hamburg, *HE* Hessen, *NI* Niedersachsen, *NW* Nordrhein-Westfalen, *MV* Mecklenburg-Vorpommern, *RP* Rheinland-Pfalz, *SL* Saarland, *SN* Sachsen, *ST* Sachsen-Anhalt, *SH* Schleswig-Holstein, *TH* Thüringen

Vergleicht man die Curricula der einzelnen Fächer im Sek.-I-Bereich wird deutlich, dass neben Sport nur Biologie (*n* = 28), Physik (*n* = 2) und Chemie (*n* = 1) HL-relevante Aspekte aufgreifen (Tab. 3). Anders als im Fach Sport dominieren im Fach Biologie eine HL-Förderung über Kommunikation (32,1 %, *n* = 9) und Auswertung von Informationen (28,6 %, *n* = 8). Der Bereich der Handlung bzw. gesundheitsförderlicher Maßnahmen tritt hierbei in den Hintergrund (3,6 %, *n* = 1).

**Tab. 3:** Anteil an HL-Textelementen (Health Literacy) innerhalb der nordrhein-westfälischen Curricula der Sek. I

	Zugang	Verstehen	Auswerten	Kommunikation	Entscheidung	Handlung	Ausgangslage	Soziokul. Lernen	Ges
Biologie	1	4	8	9	5	1	–	–	*28*
Chemie	–	–	1	–	–	–	–	–	*1*
Physik	–	1	1	–	–	–	–	–	*2*
Sport	1	6	6	4	2	7	–	–	*26*
Ges	*2* *(3,5* *%)*	*11* *(19,3* *%)*	*16* *(28,1* *%)*	*13* *(22,8* *%)*	*7* *(12,3* *%)*	*8* *(14,0* *%)*	*0*	*0*	*57*

### Adressierte Altersgruppen mit HL-bezogenen curricularen Inhalten

Bereits im Grundschulbereich können Textelemente zur Förderung von HL identifiziert werden. Vergleicht man Curricula des Fachs Sport von der Primarstufe bis Sek. II fällt auf, dass HL in den Curricula der Sek. I und Sek. II häufiger thematisiert wird (*n* = 25, bzw. *n* = 24) als im Primarbereich (*n* = 20, Tab. 4). Während im Primarbereich handlungsbezogene HL-Anteile dominieren (55 %, *n* = 11), soll HL im Bereich der Sek. I und II umfassender bzw. tiefgreifender berücksichtigt werden. Im Primarbereich dominiert eine spielerische und erzieherische Annäherung an gesundheitsbezogene Themen [17]. Zwischen den Curricula der Sek. I und II finden sich kaum Unterschiede hinsichtlich des jeweiligen Anteils der benannten HL-Komponenten. Eine zunehmende kognitive bzw. reflexive Berücksichtigung des Konstrukts mit zunehmendem Alter der Schüler*innen lässt sich aufzeigen. HL-bezogene Aspekte beziehen somit alle Altersgruppen von Beginn bis zum Ende der Schulzeit ein.

**Tab. 4:** Anteil an HL-Textelementen (Health Literacy) innerhalb der nordrhein-westfälischen Sportcurricula

	Zugang	Verstehen	Auswerten	Kommunikation	Entscheidung	Handlung	Ausgangslage	Soziokul. Lernen	Ges
GS	–	1	3	–	2	11	3	–	*20*
Sek. I	1	5	6	4	2	7	–	–	*25*
Sek. II	–	5	4	5	2	7	1	–	*24*
Ges	*1* *(1,4* *%)*	*12* *(17,4* *%)*	*13* *(18,8* *%)*	*9* *(13,0* *%)*	*6* *(8,7* *%)*	*25* *(36,2* *%)*	*4* *(5,8* *%)*	*–*	*69*

### Curricular vermitteltes Gesundheitsverständnis

In der Inhaltsanalyse der Curricula lassen sich sowohl Elemente eines ganzheitlichen sowie eines salutogenetischen Gesundheitsverständnisses finden. In zehn Bundesländern (62,5 %) dominiert der Handlungsaspekt von HL und somit die physische Komponente gemäß eines ganzheitlichen und biopsychosozialen Gesundheitsverständnisses. Nachfolgend können psychische Anteile identifiziert werden, sofern man hierzu kognitive Anteile im Sinne von verstehen, auswerten und entscheiden zählt. Weiterhin wird aber auch ein salutogenetisches Gesundheitsverständnis expliziert: „Orientiert am salutogenetischen Gesundheitskonzept bilden sowohl Kenntnisse zur Verbesserung der Leistungsfähigkeit durch Bewegung und Training als auch Methoden zur Stressreduktion wichtige Voraussetzungen zur ganzheitlichen Stärkung des psychosozialen Wohlbefindens“ (Beschreibung des Inhaltsfelds Gesundheit in Nordrhein-Westfalen, [16, S. 17]). HL wird demnach differenziert operationalisiert und spricht beide Gesundheitsverständnisse an.

## Diskussion

Die Inhaltsanalyse deutscher Curricula allgemeinbildender Schulen macht deutlich, dass Aspekte der Gesundheitsförderung v. a. in den Sportcurricula zu finden sind und hierbei häufig eher als Rahmung einer physischen Kompetenzförderung aufgefasst werden können. Das Schulfach bietet durch die Kombination aus physischer Aktivität und weiteren Lerninhalten einen geeigneten Zugang, Schüler*innen frühzeitig und auf eine für sie relevant erscheinende Art hinsichtlich gesundheitsbezogener Facetten zu fördern bzw. handlungsfähig zu machen [13]. Obwohl die Curricula häufig explizit [15] oder implizit [23] auf den mehrperspektivischen Ansatz verweisen, entspricht dies zumeist nicht der intendierten breit angelegten Berücksichtigung von Gesundheit im Rahmen der formulierten Kompetenzbereiche. Dennoch könnte ein handlungsdominierter Zugang vorteilhaft sein und kommt der Haltung junger und zumeist gesunder Zielgruppen entgegen. Im Sinne der pädagogischen Gesundheitsperspektive wird hier zuvorderst die Verbesserung der körperlichen und geistigen Fitness angesprochen [13]. So könnte es für Schüler*innen gemäß dieser Annahme motivierender sein, sich über diesen Zugang mit gesundheitsbezogenen Inhalten auseinanderzusetzen.

Der Sportunterricht bietet eine geeignete Ausgangsposition, um gesundheitsbezogene Lernprozesse aufzugreifen, allerdings bedarf es einer konstruktiven Rahmung und einer Balance von sportiven und unterrichtenden Elementen, damit Schüler*innen nicht nur unbewusst von den (bewegungsbezogenen) Inhalten profitieren, sondern die Gelegenheit erhalten, diese bewusst einzuordnen [18]. Vor allem das thüringische Sportcurriculum [26] bietet diesbezüglich konkrete Ansatzpunkte und greift vergleichsweise viele HL-Facetten [5] auf, die die Eigenständigkeit der Schüler*innen fördern und zumindest entsprechend der curricularen Formulierungen eine reflexive Auseinandersetzung intendieren.

Schulische Curricula spiegeln nicht zwangsläufig die Interessen- und empfundenen Relevanzbereiche Heranwachsender wider. Die Förderung von Gesundheit und HL sind Themen, die für Kinder und Jugendliche theoretisch bedeutsam sind [27]. Sie müssen aber so umgesetzt werden, dass sie für Kinder und Jugendliche praktisch bedeutsam und interessant sind. Obwohl Curricula und hierbei v. a. die Curricula des Sportunterrichts zahlreiche Anknüpfungspunkte für eine Förderung von HL-Facetten bieten, ist gleichermaßen nicht davon auszugehen, dass diese 1:1 in Schulen und im Unterricht umgesetzt werden, sondern durch einen Übertrag in die Unterrichtskonzeption der Lehrkraft interpretiert werden. Dieser u. a. durch Stibbe [24] kritisierte Aspekt der anzunehmenden Diskrepanz zwischen curricularen Vorgaben und realer Umsetzung von Inhalten durch Lehrkräfte besteht bei der vorgenommenen Analyse weiterhin als ein dem gewählten Zugang geschuldeter blinder Fleck. Liegt in der Unterrichtskonzeption einer Lehrkraft ein Fokus auf Fitness und Bewegungszeit mit einer maximal flankierenden Anbindung gesundheitsbezogener Aspekte vor, schöpfen Lehrkräfte das im Curriculum definierte Potenzial des Schulfachs nicht aus.

Durch die inhaltlichen Festschreibungen der Curricula sollen HL-bezogene Themen frühzeitig in den (Sport‑)Unterricht einbezogen werden. Demnach sollte eine Berücksichtigung gesundheitsbezogener Themen für Kinder im schulischen Kontext normal sein, wobei es nahe liegt, dass im Primarbereich durch einen stärkeren erzieherischen und spielerischen Zugang eine grundsätzliche Auseinandersetzung angebahnt werden soll.

Die Curricula spiegeln ein weitreichendes Gesundheitsverständnis wider, dass von einer ganzheitlichen Förderung mit akutem Nutzen (z. B. gesundheitsbewusste Sportgestaltung) bis zu einem langfristig angelegten Aufbau von Schutzfaktoren entsprechend eines salutogenetischen Verständnisses reicht, um beispielsweise eine aktive Stressreduktion zu ermöglichen [1, 7]. Die breit angelegte Berücksichtigung von Gesundheit und Bereichen der HL erhöht somit die Wahrscheinlichkeit, dass Schüler*innen im Verlauf ihrer Schulzeit für sie interessante Facetten identifizieren und somit für sich profitieren können.

## Fazit für die Praxis

- Schulischer Unterricht sollte gesundheitsbezogene Inhalte explizit aufgreifen und in das Zentrum des Unterrichts stellen. Die Inhalte sind didaktisch und methodisch so aufzubereiten, dass sie für die Lebenswelt von Heranwachsenden interessant und relevant sind.
- Sportlehrkräfte sollten das curricular verankerte Potenzial ihres Unterrichts ausnutzen, indem sie Schüler*innen mit einer gesundheitsbezogenen Perspektive in die Gestaltung des Unterrichts einbinden. Am Beispiel Ausdauerlauf (Leichtathletik) kann dies mit einer Literaturrecherche der Schüler*innen zur Steigerung der aeroben Ausdauer aus einer gesundheitsbezogenen Perspektive beginnen, gefolgt von einem Austausch über die Quellenqualität, die Gestaltung einer gesundheits- und leistungsförderlichen Unterrichtseinheit, die Identifikation und der Umgang mit Anzeichen übermäßiger physischer Beanspruchung (z. B. Prävention statt Schmerzmittelnutzung) und Ideen zur dauerhaften und für Heranwachsende attraktiven Umsetzung im Alltag.
